# Mothers' Depression, Anxiety, and Mental Representations After Preterm Birth: A Study During the Infant's Hospitalization in a Neonatal Intensive Care Unit

**DOI:** 10.3389/fpubh.2018.00359

**Published:** 2018-12-07

**Authors:** Carmen Trumello, Carla Candelori, Marika Cofini, Silvia Cimino, Luca Cerniglia, Marinella Paciello, Alessandra Babore

**Affiliations:** ^1^Department of Psychological, Health and Territorial Sciences, Università degli studi G.D'Annunzio Chieti Pescara, Chieti, Italy; ^2^Department of Dynamic and Clinical Psychology, Faculty of Psychology, Sapienza University of Rome, Rome, Italy; ^3^Università Telematica Internazionale Uninettuno, Rome, Italy

**Keywords:** neonatal intensive care unit, mothers, prematurity, maternal representations, depression, anxiety

## Abstract

**Aim:** This paper aimed to explore psychological functioning and mental representations in mothers of preterm infants during the child's hospitalization in a Neonatal intensive care unit (NICU).

**Methods:** A sample including 62 mothers of premature infants (gestational age < 37 weeks) was recruited in a NICU. According to the gestational age at the time of delivery, we considered two groups: Group A included mothers whose children were born before 32 weeks of pregnancy; Group B included mothers whose children were born at or after 32 weeks of pregnancy.

Within one week of childbirth, mothers were administered two self-report questionnaires: the Edinburgh Postnatal Depression Scale (EPDS) and the State-Trait Anxiety Inventory (STAI). When their infants' medical conditions became stable, the Clinical Interview for Parents of High-Risk Infants (CLIP) was administered to mothers.

**Results:** The results showed high levels of depression and anxiety in both groups of mothers, with higher state anxiety scores in Group A than Group B. Besides, a series of hierarchical regression analyses were conducted with STAI, EPDS, and gestational age as predictors on the CLIP scores. Results indicated that EPDS scores predicted CLIP scores on parental self-image, support system, and readiness for discharge (*p* < 0.001); moreover, the interaction among depression, anxiety, and gestational age predicted the CLIP dimension of feeling of mutual recognition (*p* < 0.005).

**Conclusions:** These findings suggested that a premature birth and the child's hospitalization might exert a negative effect on the mothers' emotional state, their perception of parental self-image and, consequently, the early bond with the child—independent from the infants' gestational age at the time of the preterm delivery. The data underlined the importance of involving NICU nurses and clinicians in order to optimize the care for mothers immediately after the preterm birth and during the infant's hospitalization, taking into account psychological needs of mothers of both very preterm and moderately preterm infants.

## Introduction

Preterm birth is an important issue in public health and is a major part of worldwide neonatal mortality and morbidity (1). Research has shown that premature birth is a distressing event for parents that often report symptoms of post-traumatic stress for several years (2, 3). Latva et al. (4) have shown that even 5 or 6 years after the preterm birth, mothers might have negative views of their perinatal or postnatal period. Otherwise, it is reported that mothers with positive experiences after a preterm birth have a more effective mother-child communication than those mothers who have had negative experiences (5). Parents of preterm infants face various difficulties and sudden changes in the process of bonding with their baby. Bonding with infants begins before birth and develops after it, but if the birth occurs sooner than expected or even too early, the normal bonding process could be affected. Goldberg and Divitto (6) have demonstrated that a long stay in hospital might have a disturbing effect on the bonding process. Although in the last decade the Neonatal Intensive Care Units (NICU) have undergone some changes for facilitating the presence of parents during the hospitalization of their baby, NICU remains a stressful environment for parents, as demonstrated in many studies (7). The physical environment is characterized by monitoring equipment, tubes and wires connected to infant, noises, and chemical scents. However, the major stress experienced by parents is related to the separation from their baby and to the loss of their parental role as they had previously imagined it. As suggested by Flacking et al. (8), the feelings of separation and exclusion could be related to the lack of physical and emotional closeness which are important factors in the early relationship between parents and the newborn infant. In fact, as frequently reported in the literature, the first moments of postpartum period are fundamental for the construction of early parent-infant bonding (9, 10). During hospitalization of their baby, mothers may experience several and often contradictory emotional reactions, such as grief, sadness, guilt, fear, anger, loss of self-esteem, and sense of failure (11). In fact, this situation can be so overwhelming for mothers that they might react by emotionally distancing themselves from their children (12, 13). These emotional factors might negatively affect the mothers' ideas, thoughts, and representations about the child's appearance and behavior. In particular, mothers of preterm babies often have fewer positive ideas and expectations for their children than mothers of term babies (14, 15); these could be characterized by a communication about their child, generally positive, with specific and sensitive details about care (16). Crawford and Benoit's work (17) has shown that maternal representations could be influenced so much by traumatic events that the parent might become incapable of understanding their child's state of mind. So, when the child makes signals or expresses desires or needs, the parent might be unable to respond in a caring and appropriate way (18). As Deklyen and Greenberg's research (19) indicated, when this occurs, it constitutes a severe risk factor for mother-child relationship and for later psychopathology. Hall et al. (20) have shown that mothers, characterized by negative and unrealistic perceptions about their baby and the hospital environment, are often more intrusive, more withdrawn, and less sensitive toward the 6-month-old infants. In light of the aforementioned factors that might negatively affect the early postpartum period—considered the “sensitive time” (10) for mother-child bonding—it is very important to explore the mothers' emotional experience after a premature birth and during the hospitalization in the NICU. For this purpose, a useful tool that specifically explores parents' experience in NICU is the Clinical Interview for Parents of High-Risk Infants [CLIP; (21)]. The CLIP allows parents to reflect on and express their feelings and concerns; it could be useful to analyze the maternal representations after a preterm delivery and to detect early disruptions in the mother-infant relationship at the nursery (5, 22). Several studies have investigated the psychological symptoms in mothers of premature infants in terms of the symptoms of depression and anxiety. In fact, mothers of premature infants generally show higher rates of postpartum depression than mothers of full-term infants (23, 24). In literature, there is a broad consensus that early depressive symptoms of mothers have a negative effect on their relationship with the infant and on their parenting role, especially after a preterm birth (25). Mothers with depressive symptoms show negative perceptions of themselves, their baby, and their relationship (26, 27). Although most studies about the effects of mothers' postnatal mood on child development focus above all on postpartum depression, in the last decades, researchers have found that postpartum anxiety has independent effects, just as postpartum depression (28). In case of premature birth, mothers show high levels of anxiety symptoms (24, 29) that might compromise the maternal functions and the mother-infant interactions (30, 31). Besides, as Lotterman et al. (32) noticed in their recent study, a lot of research that explores psychological symptoms (including depression and anxiety) and the experience of mothers with premature infants in NICU, focuses mainly on very preterm gestational age range. Compared to this field of research, studies on moderate-to late preterm infants are less, although this gestational age range characterizes a high percentage of preterm births. Moreover, as far as we know, a few studies have compared maternal representations of moderately preterm and very preterm infants in the NICU during the first moments after birth. Despite the outcomes of very preterm birth are increasingly acknowledged, less attention is given to parents of moderately preterm infants. Furthermore, it remains unclear which specific factors could be most predictive of the quality of maternal representation in the NICU. Starting from the above considerations, our study aimed to more deeply explore the maternal and emotional experience in terms of mental representations, as reflected in the CLIP interview, considering depression, anxiety symptoms, and gestational age at the time of delivery.

## Materials and Methods

### Objectives

This paper overall aimed to explore differences in psychological functioning and mental representations of the infant and themselves as parents between mothers of moderately preterm infants and mothers of very preterm infants. In order to differentiate the two groups, we have referred to recent research that set the turning point at 32 weeks of gestational age (33, 34).

In particular, the current study considered two groups: Group A included mothers whose children were born before 32 weeks of pregnancy (very preterm); Group B included mothers whose children were born at or after 32 weeks of pregnancy (moderately preterm). The study had the following specific objectives:

- To verify whether Group A and Group B differ with regards to the possible presence of anxiety and depressive symptoms in mothers;
- To verify whether Group A and Group B differ with regards to the mothers' representations about the delivery and their relationship with the premature child;
- To verify whether the mothers' anxiety and depressive symptoms predict the quality of their representations of the child and of themselves as parents, considering gestational age.

### Participants

Our study is part of a longitudinal project in which mothers and fathers of preterm infants were followed since the hospitalization in NICU till up to 2 years post-partum.

The participants were 62 mothers of preterm babies born before 37 weeks of gestation recruited in NICU of the Chieti University Hospital with the Director's, pediatricians', and nurses' collaboration. Inclusion criteria were: absence of child's genetic illnesses, neonatal deformities, and neurological damages clinically identifiable at birth, mother's age at least 18-year-old, mother's good knowledge of the Italian language, and absence of mother's drug or alcohol addiction.

Maternal and infants' basic characteristics are shown in Table 1. All parents were Caucasian and most (79%) were of middle socio-economic status [SES; (35)]. A majority (95%, *N* = 59) of the parents lived together, 80.6% (*N* = 50) of the mothers were employed, and 69.4% (*N* = 43) of the children were firstborn. The mean age of the mothers was 33.98 years (standard deviation [sd] = 4.76). The children were 45% (*N* = 28) males and 55% (*N* = 34) females.

**Table 1:** Maternal and infant characteristics at NICU (*N* = 62).

**Characteristics**	**Frequency (%)**	***M***	***SD***
**MOTHERS**			
Age		33.98	4.76
Education (Years)		14.62	3.30
Middle school	8 (13.6%)		
High school	34 (54.6%)		
University	20 (31.8%)		
Maternity			
Primipara	43 (69.4%)		
Multipara	19 (30.6%)		
Currently Employed			
Yes	50 (80.6%)		
No	12 (19.4%)		
Marital Status			
Married or cohabitating	59 (95%)		
Not married or cohabitating	3 (5%)		
**INFANTS**			
Gender			
Male	28 (45%)		
Female	34 (55%)		
Gestational Age (In Weeks)		32.28	2.352
< 32 weeks	40 (35.5%)		
≥ 32 weeks	22 (64.5%)		
Birth weight (gr)		1685.42	525.394

### Procedures and Measures

Within one week after the childbirth, a group of trained psychologists administered the mothers a demographical and anamnestic form and two self-report questionnaires: Edinburgh Postnatal Depression Scale [EPDS, (36)], State-Trait Anxiety Inventory [STAI, (37)].

The EPDS is a uni-dimensional self-reported checklist, designed as a screening tool for identifying mothers at risk for post-partum depression in community settings. Subjects were asked to rate their symptoms in the past seven days on one of four response categories ranging from “0” = “not at all” to “3” = “most of the time/quite often.” The possible scores, after reversing all positive-worded items, ranged from 0 to 30 with a higher score reflecting a higher risk for post-partum depression (PPD). In the present paper, we used the Italian validated version and its related cut-off, i.e., 8/9 (38).

The STAI is a self-report anxiety behavioral instrument consisting of two separate 20-item subscales that measure trait (baseline) and state (situational) anxiety in adults. The STAI trait subscale measures relatively stable individual differences in their proneness to anxiety (i.e., differences in the tendency to experience anxiety), and the STAI state subscale measures transitory anxiety state (i.e., subjective feelings of apprehension, tension, and worry that vary in intensity and fluctuate based on the situation). In this paper, we used the Italian validated version and its related cut-off, i.e., 39/40 (39).

For the second step, when the infants' medical conditions were stable, a clinical interview [CLIP, (21)] was administered to the mothers by a second group of psychologists who were blind to its administration and the results of self-report questionnaires.

The CLIP was originally developed by the authors to assess the feelings and perceptions of preterm children's parents. It consists of a semi-structured interview allowing the clinician to extensively explore the parental experience. Further, its flexibility allows the clinician to adapt the questions according to the conversational flow, to better explore the parent's emotional experience. This interview requires about 1 h to be completed and addresses eight main areas: infant's current condition, pregnancy course, labor and delivery, relationship with the baby and feelings as a parent, reactions to NICU environment and staff, relationship with the family and social support, discharge and beyond, and a final wrapping up.

The CLIP is audio-recorded and transcribed verbatim; the authors recommended coding the interview through a content analysis; afterwards, Keren et al. (5) developed a coding system to analyze two areas: “readiness for parenthood” and “parental rejection.”

Clinical interviews were administered in an empathetic and understanding environment. The interviews lasted an average of 1 h and were audio-recorded with the mothers' permission and subsequently transcribed verbatim, as previously indicated by the authors.

After administering all the measures, two sub-groups were created on the basis of gestational age (33, 34, 40, 41): Group A (*very preterm*) included mothers whose children were born between 28 and 31 weeks; Group B (*moderately preterm*) included mothers whose children were born between 32 and 36 weeks of pregnancy.

Participation in the study was voluntary. All the participants received a letter containing detailed information on the main aims of the study and signed a written informed consent. The questionnaires were alphanumerically coded in order to guarantee anonymity. In the current observational study any diagnostic process was performed; in addition, it involved women who had no history or ongoing evidence of any psychiatric illness; hence, we believe that the approval of the study by the Ethics Committee was not appropriate. Nevertheless, an additional opinion was asked to the Psychological Review Board of our Department. The Board found that all the employed procedures and measures were fully compliant with the Ethics Code of the Italian Board of Psychology— the national authority that provides ethical guidelines for research and clinical practice.

### Data Analysis

Descriptive analyses attested that all variables were normally distributed. The analyses of variance (ANOVAs) were used to test significant differences between Group A and Group B scores on STAI, EPDS, and CLIP, and Bonferroni's *post hoc* tests were applied. The calculated *p*-values are reported with their respective F statistics and degrees of freedom (df), with values < 0.05 being considered significant. Mean values are reported with standard deviations (sd_s_). Finally, a series of hierarchical regression analyses were conducted to investigate the influence of specific anxious and depressive symptoms (STAI and EPDS) on the mothers' representations about the delivery and their relationship with the premature child (CLIP). In all the analyses we conducted, the child's gender and mothers' age showed no significant effect on the variables. All analyses were performed on the SPSS software (Version 18.0).

## Results

### Differences in Group A and Group B Scores on the Symptoms of Anxiety and Depression

An ANOVA conducted on Group A and Group B scores on STAI-Y1 showed a group effect [F_(1, 60)_ = 7.418; *p* < 0.01). *Post-hoc* analysis showed that Group A has significantly higher scores than Group B on STAI-Y1 (State Anxiety) (*p* < 0.01); 72% of the subjects in Group A and 45% in Group B exceeded the clinical cut-off for STAI-Y1 for the Italian population (39).

An ANOVA conducted on Group A and Group B scores on STAI-Y2 showed no group effect [F_(1, 60)_ = 0.647; *p* > 0.5]. Although no statistically significant differences were found between the two groups on STAI-Y2 (Trait Anxiety), 36% of subjects in Group A, and 37.5% in Group B exceeded the clinical cut-off for STAI-Y2 for the Italian population (39).

An ANOVA conducted on Group A and Group B scores on EPDS showed no group effect [F_(1, 60)_ = 0.66; *p* > 0.5]. Although no statistically significant differences were found between the two groups on EPDS, 68% of subjects in Group A and 60% in Group B exceeded the clinical cut-off for EPDS for Italian population (38).

Table 2 shows mean scores and standard deviations values.

**Table 2:** Mean scores and standard deviations [M(sd)] values of STAI-Y1, STAI-Y2 and EPDS in Group A and Group B.

	**STAI-Y1**	**STAI-Y2**	**EPDS**
Group A	48.55 (13.68)	38.27 (8.64)	13.05 (5.25)
Group B	42.85 (12.69)	37.21 (10.36)	11.33 (5.78)
*p*	< 0.01	n.s	n.s

### Differences in Group A and Group B Scores on the Mothers' Representations About the Delivery and Their Relationship With the Premature Child

An ANOVA conducted on Group A and Group B scores on CLIP showed a group effect on several dimensions (*p* < 0.05). *Post-hoc* analysis showed that Group A had significantly higher scores than Group B on CLIP dimension of: perceived infant's current condition (F_(1, 60)_ = 0.016; *p* < 0.05), first feelings toward baby (F_(1, 60)_ = 0.035; *p* < 0.05), readiness for discharge (F_(1, 60)_ = 0.003; *p* < 0.05), and organization of content (F_(1, 60)_ = 0.016; *p* < 0.05). No other statistically significant difference was found on the other CLIP subscales (*p* > 0.5). Table 3 shows mean scores and standard deviations values.

**Table 3:** Mean scores, standard deviations and *p*-values on CLIP main areas and subscales in Group A and Group B.

**CLIP main area**	**CLIP subscale**	**Group A**	**Group B**	***p***
Infant's current condition		0.68 (1.68)	0.35 (0.70)	< 0.05
Pregnancy	First reaction to pregnancy	1.32 (0.65)	1.18 (0.45)	n.s
	Planned pregnancy	1.45 (0.51)	1.38 (0.49)	n.s
Course of pregnancy	Physical and/or emotional complications	2.05 (1.13)	2.48 (1.24)	n.s
	Timing of “pregnancy feeling real”	2.14 (1.11)	1.97 (1.12)	n.s
Labor and delivery	Readiness for delivery	2.45 (0.60)	2.41 (0.64)	n.s
	Fear of loss during delivery	2.32 (0.65)	2.05 (0.57)	n.s
Relationship with baby and feelings as a parent	First feelings toward baby	1.55 (0.60)	1.20 (0.41)	< 0.05
	Present feelings toward baby	1.33 (0.48)	1.15 (0.36)	n.s
	Feeling of mutual recognition	1.52 (0.68)	1.46 (0.55)	n.s
	Parental self-image	1.33 (0.58)	1.33 (0.53)	n.s
Reaction to NICU	Reaction to staff	1.43 (0.51)	1.32 (0.53)	n.s
	Reaction to NICU setting	1.67 (0.62)	1.93 (0.92)	n.s
Support system		1.32(0.48)	1.30 (0.46)	n.s
Discharge and beyond	Foreseen future for baby	1.50 (0.67)	1.23 (0.43)	n.s
	Readiness for discharge	1.70 (0.70)	1.24 (0.43)	< 0.05
Affect		1.68(0.65)	1.53 (0.56)	n.s
Organization of content		1.36(0.58)	1.26 (0.44)	< 0.05
Richness of content		1.62(0.59)	1.46 (0.60)	n.s

### Impact of Mothers' Anxiety and Depressive Symptoms on the Quality of Their Representations of the Child and of Themselves as Parents, With Respect to the Gestational Age

A series of hierarchical regressions have been conducted with STAI, EPDS, and gestational age as predictors on the CLIP scores. The results showed that EPDS scores predicted CLIP scores on parental self-image, support system, and readiness for discharge (*p* < 0.01); gestational age predicts scores on the CLIP main area of course of pregnancy (*p* < 0.05); STAI-Y1 scores, interacting with gestational age, predict the CLIP main area of affect (*p* < 0.01). EPDS, interacting with STAI-Y1, STAI-Y2 scores, and gestational age, predict the CLIP subscale scores of feeling of mutual recognition (*p* < 0.05).

Table 4 shows results and values of the regression analyses (significant results only).

**Table 4:** Results and values of the regression analyses (significant results).

**STAI/EPDS/gestational age**	**CLIP main areas and subscales**			
	**R2**	**ß**	**t**	***p***
EPDS	Parental self-image			
	0.061	0.671	29.431	< 0.001
	Support system			
	0.031	0.767	32.123	< 0.001
	Readiness for discharge			
	0.045	0.369	3.475	< 0.001
Gestational age	Course of pregnancy			
	0.069	0.537	2.465	< 0.05
STAI-Y1∙ gestational age	Affect			
	0.052	0.613	2.324	< 0.001
EPDS∙ STAI-Y1∙ STAI-Y2 ∙ gestational age	Feeling of mutual recognition			
	0.089	0.413	2.328	< 0.005

*∙Scores in association with*.

## Discussion

Premature delivery and the subsequent hospitalization in NICU are considered early adverse experiences, which could affect the emotive states of mothers, their mental representations, their perceptions of infants, and their relationship in the early postpartum moments, that are assumed to be significant for maternal bonding to the infant.

Our study aimed to explore the maternal emotional states in NICU, with reference to anxiety and depressive symptoms, mental representations, and gestational age at the time of delivery.

With regard to the first aim, results showed that mothers of premature babies experience high levels of psychological distress in both the investigated groups, namely very preterm (Group A) and moderately preterm (Group B) children.

In reference to symptoms of anxiety, the mothers' scores significantly differed between the two groups. More specifically, the State Anxiety seemed to be the only one influenced by the baby's gestational age, in fact mothers in Group A showed higher anxiety levels than those in Group B.

These findings are consistent with the previous studies reporting that mothers of very preterm infants may be more concerned and worried about their babies' survival as compared to those of moderately preterm infants (42, 43).

On the contrary, there are no differences in Trait Anxiety levels when the two groups were compared, despite a large number of mothers of the whole sample exceeding the clinical cut-off for the Italian population (39). This result is in line with other ones that highlight elevated anxiety symptoms following a premature delivery (44, 45). An alternative explanation of the high levels of state anxiety in our sample may not exclude a post-traumatic state of the mothers, following the very preterm delivery. In fact, several recent studies found that posttraumatic stress represented the most common reactions after a premature childbirth (46, 47). However, this hypothesis could be more suitably explored in future research using the STAI alongside with other more specific tools for post-traumatic stress disorder.

If we move to consider depressive symptoms, contrary to general expectations, we do not find significant differences between the two groups, despite a high percentage of mothers exceeding the clinical cut-off for EPDS for the Italian population (38), regardless of the gestational age at the time of delivery. In our sample, during the infant's NICU hospitalization, maternal depressive symptoms in both the groups were equally elevated which was in line with other studies (48–51).

We may hypothesize that, in case of preterm birth, the precarious conditions of the child, the cold and sterile environment of the NICU, and the ambiguity of maternal role in the hospital setting make the mothers more vulnerable to depressive symptoms, even in the case of moderately preterm infants.

The mothers' feelings of helplessness, exclusion, and alienation could further increase the level of distress and might thus impact their transition to motherhood (52, 53). In fact, previous studies showed that the prevalence of postpartum depression after premature delivery can be estimated up to 70% (54–56).

Our finding of higher levels of anxiety and depressive symptoms in mothers of preterm infants is consistent with other research that explored the distress in mothers of premature infants as compared to mothers of full-term infants (31, 57, 58).

In the literature, few studies have examined the level of symptoms immediately after birth or during NICU hospitalization. The current study has estimated maternal symptomatology during the infant's hospitalization, 1 week after delivery. Our results showed that beyond the infant's gestational age, mothers present a high risk of anxious and depressive symptomatology. Therefore, it could be important to pay close attention to mothers' emotional experiences related to premature birth since the first moments after delivery.

Since the first contact between a mother and her child takes place inside the NICU, it is very important to provide the mothers with psychological support or assistance right from birth in order to ensure their well-being and prevent future problems.

Another purpose of this research was to deeply explore the maternal representations during the NICU stay. More specifically, with regard to the second aim, mothers of very preterm infants differ from mothers of moderately preterm infants only in four areas of maternal representations, as reflected in the CLIP. Specifically, as compared to the mothers of moderately preterm infants, mothers of very preterm babies showed more negative experiences and perceptions relating to their infants and their relationship with them. In fact, these mothers were characterized by a greater “fear of loss of the baby” (a dimension related to the area of maternal perception of the child's current condition) and more negative “first feelings toward the baby.” Additionally, they did not feel ready for discharge and the narratives of their representations were disorganized.

These major difficulties of mothers of very preterm children in the narrative organization of representations, their negative feelings toward the infant, and the low confidence in their role could be related to the perception of greater vulnerability of their baby, that increases their fear of loss (59). Seeing their infants as fragile and in danger in the NICU is very stressful for mothers, and it may generate an “emotive crisis” (7, 60).

The mothers of preterm infants often show feelings of ambivalence about their relationship with their child and feelings of unreality of “being mothers” during the NICU stay (61). In fact, in the case of very preterm infants, mothers spend more time in the NICU, where they are continuously in touch with the experience of the infants' fragility and risk of mortality (62). Living in a state of psychological and physical separation from their babies is intensified by the artificial environment of the NICU. All these early and difficult experiences could affect the development of maternal mental representations.

Finally, in reference to the third objective of the study, according to our results, depressive symptoms were the strongest predictors of the quality of maternal representations of the child and of themselves as mothers and of the child. In particular, depression seemed to predict more areas of representations with respect to the other variables we had considered. It predicted an insecure parental self-image, negative perception of support system, and lower readiness for discharge (this last area investigated the mothers' impression and expectations about homecoming and the baby's future development).

Generally, premature birth is considered a stressful and potentially traumatizing event (63) followed by the hospitalization in the NICU, where there is a prolonged separation between the mother and the infant. This situation might generate feelings of depression and the mothers' poorer psychological well-being which may lead to lesser psychological investment in relationship with the infant (15) and altered perception of both the mothers' parental role and the support system.

In addition, our findings showed that depressive symptoms, in interaction with anxiety (Trait and State) and gestational age, predict the CLIP area “feeling of mutual recognition,” regarding the mothers' perception of being recognized by their children in their parental role. It could be concluded that in presence of the comorbidity of anxiety and depression, the lower the gestational age at the birth, the less the mothers feel to be recognized by their infants.

As suggested by Feldman (64), close proximity, nurturing, and interaction with the baby play an important role for the early mother-child interaction, consolidating mothers' confidence in her parenting role. In the NICU, these conditions are absent: the early separation between the mothers and their infants, the loss of maternal role (60), the feeling of responsibility for the unhealthy state of the infants (65), and negotiation of the infant's care with nurses and medical staff within the unit (66) are associated with a higher risk of long-term psychological problems, such as depression, anxiety, feelings of isolation, and fear for the child's well-being (67). These emotive and psychological states, with a lower gestational age, that usually requires prolonged hospitalization, seem to influence above all the aspect concerning “recognition” and “reciprocity.” In fact, physical closeness represents the prerequisite for early parent-to-infant bonding for maternal behaviors and for reciprocity between the mother and her child (64, 68). These data are in line with other research that underlined that the NICU stay could hinder the development of the intuitive parental capacities in the mothers of very preterm infants (69, 70).

Further research is needed to explore the mechanism behind the development of maternal representations in the particular situation of premature birth. Indeed, several studies have demonstrated that maternal representations influence the way in which a mother interacts with her baby (71).

Overall, our research highlights some important aspects of mothers' experience and their emotional state in the early moments of the child's life during the hospitalization in NICU in a situation of high risk for the infant.

Nevertheless, our study has some limitations, such as the small sample size and its recruitment in only one NICU; this may limit the generalizability of the results. In addition, we did not consider a comparison group of mothers with full-term children to test depressive and anxiety levels. Hence, future research could try to replicate these findings using larger and more diversified samples, also referring to non-Italian mothers, given the wide cross-cultural variations in maternal reactions to preterm delivery.

However, the present study makes a relevant contribution to knowledge regarding the emotional state of the mothers of premature babies, highlighting a difficult emotional situation not only for the mothers of very preterm babies but also for those of moderately preterm babies. Often, it happens that high-risk mother-infant dyads receive more psychological attention than low-risk ones (5). To improve care, it is very important to also understand the experiences of these mothers who are at the risk of being neglected. In addition, our study integrated different tools (questionnaires and interviews) and the use of a clinical interview tool— the CLIP— built specifically for parents of premature babies, that allowed us to extensively analyze maternal representations, retracing the path from pregnancy to experience in NICU with the mothers. Undeniably, not prematurity in itself but the presence of certain emotive states, negative thoughts, and perceptions in parents might be indicative of the difficulties in parent-infant relationship (72).

Parenting interventions in the NICUs play an important role in facilitating the bonding between the mother and the infant, providing support for these vulnerable families. Benzies et al. (73), in their meta-analysis, showed that early interventions in NICU decreased maternal anxiety and depressive symptoms and increased the mothers' sense of self-efficacy.

In fact, although NICU's primary function is medical assistance for infants, it is also the place where there is the first mother-child encounter and where all the early dynamics of their relationship begin. For this reason, it is crucial to conduct further research in this area.

## Author Contributions

All authors listed have made a substantial, direct and intellectual contribution to the work, and approved it for publication.

### Conflict of Interest Statement

The authors declare that the research was conducted in the absence of any commercial or financial relationships that could be construed as a potential conflict of interest.

## Abbreviations

CLIP: Clinical Interview for Parents of High-Risk Infants
EPDS: Edinburgh Postnatal Depression Scale
NICU: Neonatal Intensive Care Unit
STAI: State-Trait Anxiety Inventory.
